# Is isolated systolic hypertension an indication for renal denervation?

**DOI:** 10.3389/fphys.2014.00505

**Published:** 2014-12-19

**Authors:** Yutang Wang

**Affiliations:** School of Applied and Biomedical Sciences, Federation University AustraliaMount Helen, VIC, Australia

**Keywords:** blood pressure, clinical trials, isolated systolic hypertension, renal artery stenosis, renal denervation

Ewen et al. recently reported in the journal *Hypertension* that they investigated, for the first time, the effect of renal denervation on blood pressure in 63 patients with isolated systolic hypertension (Ewen et al., 2014). The authors concluded that renal denervation reduced office and ambulatory blood pressure in patients with isolated systolic hypertension (Ewen et al., 2014). However, this conclusion may not be drawn, as renal denervation may not decrease ambulatory blood pressure in these patients. The potential risk of renal denervation may overweigh its benefit in patients with isolated systolic hypertension. Therefore, adjusted drug treatment may be recommended to these patients before renal denervation.

Ambulatory blood pressure monitoring is the gold standard to diagnose true hypertension and removes the white coat effect (Hermida et al., 2013). Ambulatory blood pressure is superior to office blood pressure in predicting cardiovascular events (Staessen et al., 1999) and mortality (Dolan et al., 2005). The 24-h ambulatory systolic blood pressure in these 63 patients in Ewen et al.'s report decreased by 8 ± 8 and 7 ± 8 mm Hg at 6 and 12 months respectively after renal denervation. However, this study lacked a control group as the authors pointed out as a limitation. It has been reported that the sham procedure reduced 24-h ambulatory systolic blood pressure by 5 ± 15 mm Hg at 6 months (Bhatt et al., 2014). Therefore, compared with the sham procedure, renal denervation may not decrease ambulatory blood pressure in those patients with isolated systolic hypertension.

Consequently, the risk posed to patients with isolated systolic hypertension by renal denervation may overweigh the minimal benefit of renal denervation via lowering blood pressure. For example, renal artery stenosis after renal denervation is of concern. The renal artery stenosis rate in the Symplicity HTN trials (*N* = 45, 106, and 535 for the Symplicity HTN-1, HTN-2, and HTN-3 trials, respectively) ranges from 0.3 to 2.2% (Krum et al., 2009; Esler et al., 2010; Bhatt et al., 2014). However, more and more studies with a smaller sample size (Worthley et al., 2013; Versaci et al., 2014) and case reports (Kaltenbach et al., 2012; Vonend et al., 2012; Aguila et al., 2014; Bacaksiz et al., 2014; Chandra et al., 2014; Pucci et al., 2014) showed relatively higher rates of development or progression of renal artery stenosis after renal denervation. Ewen et al. did not observe any hemodynamically significant renal artery stenosis in these 63 patients with isolated systolic hypertension within 12 months (Ewen et al., 2014). However, ultrasonography, which was used by the authors, has limitations in detecting renal artery stenosis (Zhang et al., 2009; Lao et al., 2011).

Renal denervation is regarded as a last resort for patients with resistant hypertension (Persu et al., 2012). It is reported that about 9% of adults with hypertension suffer from resistant hypertension (Persell, 2011), which is often defined as elevated blood pressure despite treatment with at least 3 antihypertensive agents including a diuretic at maximal tolerated or highest recommended doses (Bohm et al., 2014). The prevalence of resistant hypertension is likely over-estimated due to drug non-adherence. For example, blood pressure in 20 of 65 patients with resistant hypertension was normalized after witnessed intake of antihypertensive drugs (Fadl Elmula et al., 2014). Resistant hypertension has been classified as “true” resistant hypertension if blood pressure is still elevated after witnessed intake of antihypertensive drugs (Fadl Elmula et al., 2014). Blood pressure in some patients with “true” resistant hypertension could be controlled by adjusted drug treatment. For example, Fadl Elmula et al. reported that adjusted drug treatment significantly decreased ambulatory systolic blood pressure from 152 ± 12 mm Hg at baseline to 133 ± 11 mm Hg at 6 months in 10 patients with “true” resistant hypertension (Fadl Elmula et al., 2014). In addition, adjusted drug treatment lowered ambulatory systolic blood pressure to below 135 mm Hg in 7 of these 10 patients with “true” resistant hypertension (Fadl Elmula et al., 2014). Therefore, patients with isolated systolic hypertension may be offered with adjusted drug treatment before being offered with renal denervation.

In summary, renal denervation may not decrease ambulatory blood pressure in patients with isolated systolic hypertension. Adjusted drug treatment may be recommended to these patients before renal denervation, as the risk might overweigh the benefit of renal denervation in these patients.

## Conflict of interest statement

The author declares that the research was conducted in the absence of any commercial or financial relationships that could be construed as a potential conflict of interest.
